# Acute effects of moderate-intensity continuous and high-intensity interval exercise on vascular function

**DOI:** 10.1007/s00421-026-06392-6

**Published:** 2026-08-08

**Authors:** Simon Iskra, Gašper Turnšek, Armin Paravlić

**Affiliations:** 1 Faculty of Sport, University of Ljubljana, Gortanova 22, Ljubljana, 1000 Slovenia; 2 Science and Research Centre Koper, Institute for Kinesiology Research, Koper, Slovenia; 3 Faculty of Sport Studies, Masaryk University, Brno, 625 00 Czech Republic

**Keywords:** Aerobic exercise, Vascular function, Arterial stiffness, Microvascular reactivity

## Abstract

**Purpose:**

To assess and compare the acute effects of high-intensity interval training (HIIT) and moderate-intensity continuous training (MICT) on central and peripheral arterial stiffness, as well as endothelial and microvascular reactivity in healthy, physically active adults.

**Methods:**

Sixteen healthy young adults (8 M/8F; age: 24 ± 3 yrs) completed a randomized crossover trial consisting of two cycling exercise protocols: HIIT (4 × 4 min at 100–105% respiratory compensation point (RCP) power) and MICT (30 min at 65% RCP power). Arterial stiffness was assessed via carotid-femoral pulse wave velocity (cfPWV) and brachial-radial pulse wave velocity (brPWV). Flow-mediated slowing (FMS) and microvascular reactivity following a vascular occlusion test were used for determination of vascular reactivity. All measurements were taken within 5–15 min following exercise cessation.

**Results:**

A significant time-by-condition interaction was observed for brPWV (F = 7.08, *p* = 0.024), revealing a significant increase following MICT whereas no significant change was observed following HIIT (MD = 0.35 m/s). There were no interactions regarding cfPWV, although a trend towards increased cfPWV following both conditions was observed (F = 3.42, *p* = 0.086), as well as mean arterial pressure (F = 4.03, *p* = 0.063). No significant changes were observed for FMS or microvascular reactivity.

**Conclusion:**

These findings demonstrate a differential acute vascular response to exercise intensity. MICT elicited a significant increase in brPWV following MICT, while no change was observed following HIIT. Central arterial stiffness response does not seem to be mediated by exercise intensity. These distinct, segment-specific responses highlight the importance of assessing both central and peripheral vascular compartments when evaluating the acute impact of aerobic exercise.

**Trial registration number (clinical trials):**

NCT07048509.

**Graphical abstract:**

**Figure:**
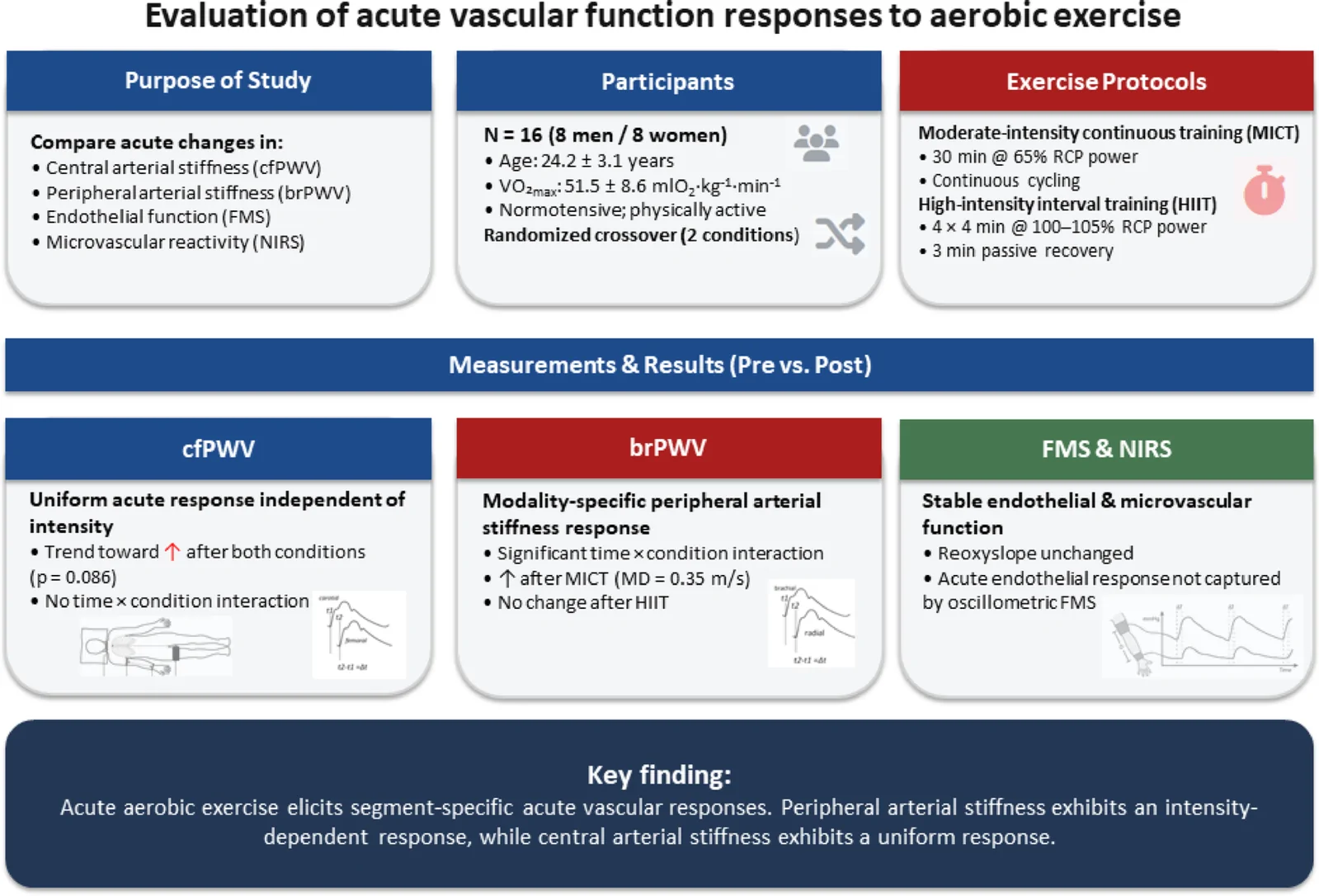

## Introduction

Aerobic exercise is a well-established strategy for cardiovascular disease (CVD) prevention and induces favourable vascular adaptations, primarily through increased shear stress and upregulation of vasoactive substances (Mancia et al. 2023). It has been shown to be more effective than resistance or combined exercise for improving vascular function, as assessed by flow-mediated dilation (FMD), with interval aerobic training demonstrating superior effects compared with continuous training (Mattioni Maturana et al. 2021; Paravlic et al. 2025).

While chronic effects of exercise on different measures of vascular function are well established, acute effects are comparably less clear. Recent meta-analyses have shown conflicting results. A meta-analysis by Saz-Lara et al. (2021) has shown that measures of arterial stiffness tend to decrease in the 5–30 min following exercise, but tend to normalise back to pre-exercise values in a 24 h period. In contrast, a meta-analysis by Pierce et al. (2018) showed no effect of aerobic exercise on central arterial stiffness. Another systematic review by Mutter et al. (2017) found increases in arterial stiffness immediately following exercise and a drop in both central and upper body-peripheral arterial stiffness in the > 5 min following the cessation of exercise. These results reveal a conflicting picture with heterogeneous results from the current studies (Heffernan et al. 2007; Rakobowchuk et al. 2009; Tordi et al. 2010), partly owing to different approaches to quantification of exercise configuration domains, including exercise duration, intensity, modality, measures of interest, population included and subsequent data analysis.

In the context of chronic adaptations, both continuous and interval exercise provide an effective stimulus for long-term CVD risk prevention, with interval exercise emerging as more effective (Paravlic et al. 2025). This difference in response might be owed to a different hemodynamic profile between the two exercise protocols. While continuous exercise induces increased laminar shear stress, and interval exercise of higher intensities provides an intermittent oscillatory peak stress with higher peak magnitudes compared to continuous exercise (Tinken et al. 2009; Shi et al. 2022). This inherent difference in the stimulus to the artery walls might lend itself to both differences in the magnitude of adaptations and the type of adaptations to the vascular wall and parts of the vasculature.

The acute changes in vascular function represent the initiation of a vascular response pattern, akin to many physiological system adaptation patterns, and may provide insight into subsequent chronic adaptations (Dawson et al. 2013). Indeed, the magnitude of acute FMD responses has been shown to correlate with long-term vascular adaptations following aerobic exercise (Dawson et al. 2018). In the present study, FMD was omitted from the measurement procedures in order to focus on the concurrent measurements of microvascular reactivity and flow-mediated slowing (FMS), which is an indirect measure of the vasodilatory response to reactive hyperaemia. Considering the research gap on the difference in acute response of different vascular function measures following aerobic exercise, as well as heterogenous results on acute response of these measures in the literature, the present study aimed to assess the acute response of central and peripheral arterial stiffness, as well as peripheral and microvascular hyperaemic response to AE. A secondary aim of this study was to investigate whether a the acute responses of the aforementioned vascular measures differ between moderate-intensity continuous training (MICT) and high-intensity interval training (HIIT) in healthy, physically active individuals. We hypothesized that HIIT would elicit more favourable acute changes in both central and peripheral measures of vascular function compared to MICT.

## Methods

### Study design and participants

Sixteen participants (8 men and 8 women) who met the predefined inclusion criteria were enrolled in this study. The study was designed as a crossover randomized controlled trial and comprised of three laboratory visits. Data collection was conducted between June 2025 and December 2025. The study was single-centre, taking place at the laboratory of the Faculty of Sport, University of Ljubljana.

Participants were recruited through mass media, and online advertisements. The study was conducted in accordance with the principles of the Declaration of Helsinki and received approval from the National Medical Ethics Committee of the Republic of Slovenia (reference number: 0120-63/2025-2711-3). Prior to participation, all individuals provided written informed consent. Participants were informed of their right to withdraw from the study at any time and without providing a reason. The study was prospectively registered at ClinicalTrials.gov (NCT07048509).

### Inclusion and exclusion criteria

During the screening visit, two researchers (SI and GT) conducted a structured interview with interested individuals. During this visit, habitual physical activity levels were assessed using the Global Physical Activity Questionnaire (GPAQ), and medical as well as family history of disease were recorded. Female participants also answered the Reproductive Status Questionnaire (RSQ) to assess their menstrual cycle characteristics (Schmalenberger et al. 2021). In addition, ambulatory blood pressure was measured. Of the 52 individuals initially screened, 16 met all inclusion criteria and were therefore enrolled in this study.

Eligible participants were adults aged between 18 and 50 years who were classified as moderately physically active (600–3000 MET∙min^− 1^∙week^− 1^) according to the GPAQ questionnaire (Armstrong and Bull 2006) and met training status levels 1 or 2 as defined by McKay et al. (2022). Furthermore, only individuals with optimal blood pressure values (systolic < 120 mmHg and diastolic < 80 mmHg) were included.

Participants were excluded if they presented with elevated blood pressure, were using antihypertensive or lipid-lowering medications, were current smokers, sustained a lower-extremity injury within the preceding six months, or had a history of chronic non-communicable diseases, established cardiovascular disease, or metabolic disorders or any other state limiting them from exercise. Additional exclusion criteria included pregnancy, absence of a regular menstrual cycle, and a body mass index ≥ 30 kg/m².

### Measurement procedures

**Fig. 1:**
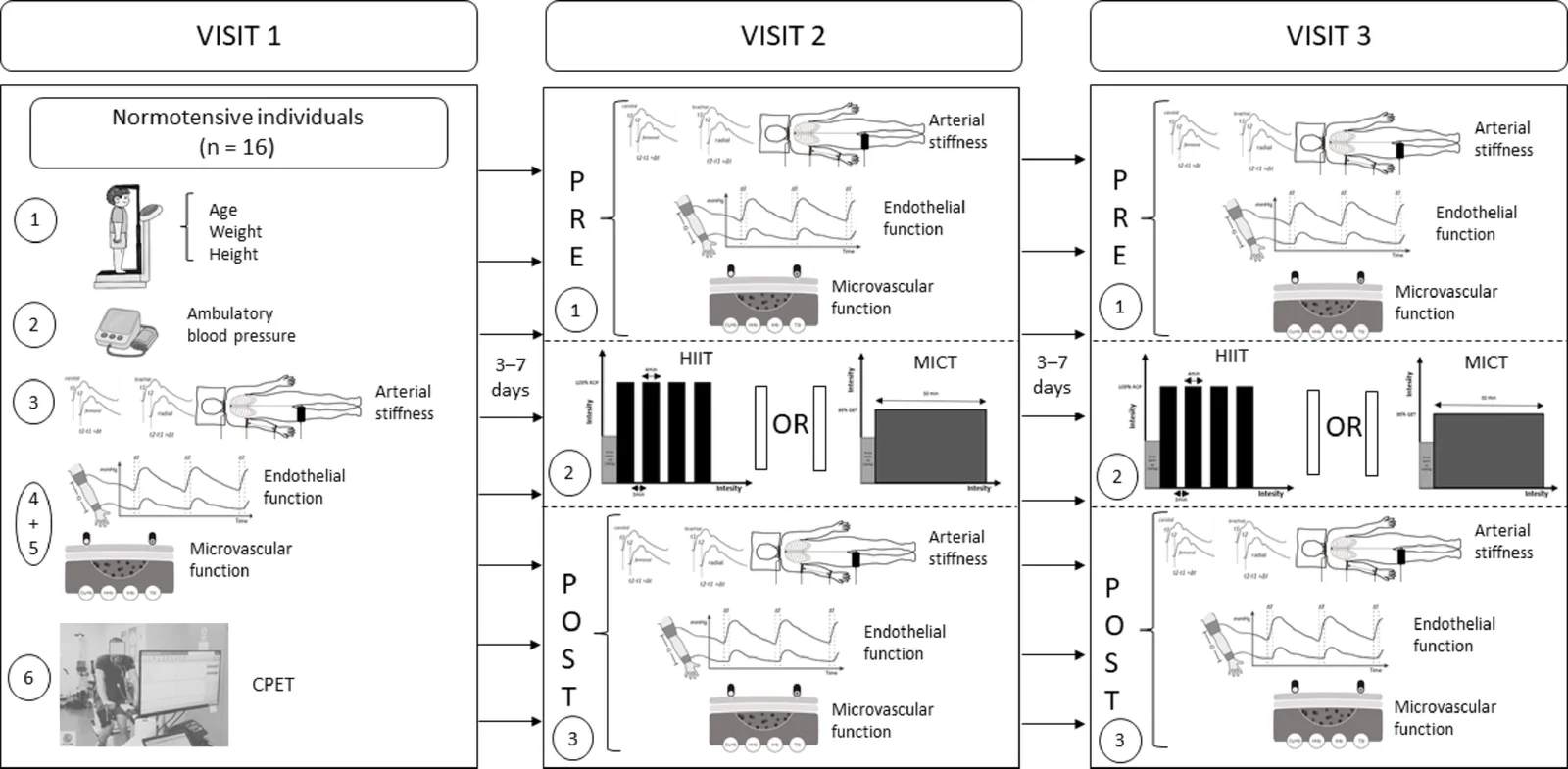
Schematic representation of the study. MICT – moderate-intensity continuous training, HIIT – high-intensity interval training, CPET – cardiopulmonary exercise testing

In addition to the screening visit, participants completed three experimental visits. The first visit was dedicated to the assessment of body composition, vascular function and the performance of a cardiopulmonary exercise test (CPET) on a cycle ergometer. Visits 2 and 3 were designed to evaluate acute vascular responses to a randomly assigned exercise stimulus, either high-intensity interval training (HIIT) or moderate-intensity continuous training (MICT) (Fig. 1). A battery of vascular function tests was performed before and after each exercise stimulus. The battery was assessed in a standardized order with measurement of pulse wave characteristics, followed by central and peripheral pulse wave velocity (PWV) measurement and finally a concurrent measurement of flow-mediated slowing (FMS) and microvascular reactivity. Following the arrival to the laboratory, the participants were instructed to sit on a chair for 10 min in a standardized position with the arm resting on a table. Afterwards, blood pressure measurements were performed in triplicate. Afterwards, the participants were instructed to lay supine on a table for an additional 15 min, allowing for haemodynamic stabilisation and standardisation of the pre-exercise measurements. Following the vascular function measurements, the participants exercised according to the exercise protocol. After cessation of exercise, a 3-minute rest in a sitting position on the cycling ergometer was employed. Afterwards the participants lay supine on a table for an additional 2 min, allowing for pressure cuff and near-infrared spectroscopy (NIRS) sensor placement. The vascular function measurements were performed in approximately 10 min, with measurements post-exercise stimulus being taken within a time window of 5–15 min post-exercise cessation to ensure measurements in a standardized time window and avoid measurements immediately following exercise. All measurements were performed in a quiet, temperature-controlled environment (22–24 °C).

Randomization of the exercise conditions for visits 2 and 3 was performed using the RAND function in Microsoft Excel, with a 1:1 allocation ratio. The visits were scheduled 3–7 days apart and conducted at the same time of day to minimize the potential influence of circadian rhythm variation.

Participants were instructed to refrain from any additional structured physical activity, as well as from alcohol and caffeine consumption, in the days preceding each of the three visits and throughout the duration of the study. Furthermore, they were advised to arrive well rested and to consume a standardized meal 2–3 h prior to each laboratory visit. Female participants were instructed to avoid conducting measurements during the menstruation phase of the menstrual cycle (Shenouda et al. 2018; Williams et al. 2020). All procedures were supervised by two investigators (GT and SI).

### Body composition

Body composition was assessed using bioelectrical impedance analysis (InBody 720, Biospace, South Korea) upon participants’ arrival at the laboratory during the first visit. Prior to body composition assessment, body height was measured using a stadiometer (GPM, Siber Hegner & Co., Switzerland), with participants standing barefoot in an upright position.

### Blood pressure

Participants were seated quietly for 10 min in a calm, temperature-controlled environment. During the measurement, the non-dominant arm was fully extended and supinated, supported on a flat surface at heart level. Blood pressure was measured using an automated upper-arm oscillometric device (M7, Omron, Japan). Three consecutive measurements were obtained at one-minute intervals, and the mean of the final two readings was used for subsequent analyses.

### Arterial stiffness and pulse wave characteristics

Pulse wave analysis was performed at the brachial artery using an oscillometric system (Vicorder, software version 4; Skidmore Medical, United Kingdom). A pneumatic cuff was positioned around the upper arm and inflated to a sub-diastolic pressure of 65 mmHg to record arterial pressure waveforms using a volume displacement technique. Central aortic pressure indices, including aortic pulse pressure (AoPP) and mean arterial pressure (MAP), were derived using a validated brachial-to-aortic transfer function embedded in the manufacturer’s software. To enhance signal reliability, at least ten consecutive waveforms were recorded and averaged for analysis.

Arterial stiffness was assessed by measuring PWV across two arterial segments: brachial–radial (brPWV) and carotid–femoral (cfPWV). For brPWV assessment, cuffs were placed around the wrist and the proximal upper arm and simultaneously inflated to 65 mmHg. The linear distance between the midpoints of the cuffs was measured directly and used for PWV calculation. For cfPWV, one cuff was positioned over the right carotid artery and a second cuff around the proximal right thigh. The arterial path length was estimated as the distance between the suprasternal notch and the midpoint of the thigh cuff, in accordance with the manufacturer’s recommendations. PWV was calculated as the ratio of the measured path length to the transit time between the proximal and distal pulse wave onsets.

### Flow mediated slowing

An oscillometric device (Vicorder, 80 Beats Medical, Germany) was used to assess brachial–radial FMS. All measurement procedures were performed by manufacturer guidelines. Briefly, two oscillometric cuffs were placed on the left upper arm and wrist and connected to the Vicorder device. The distance between the midpoints of the brachial and radial cuffs was measured and defined as the brachial–radial path length. The FMS protocol consisted of a 3-minute baseline period, followed by a 5-minute arterial occlusion period, during which the brachial cuff was inflated to 50 mmHg above the previously measured systolic blood pressure, and a subsequent 4-minute post-occlusion period. During both the baseline and post-occlusion phases, the brachial and radial cuffs were inflated to a sub-occlusive pressure (65 mmHg) to allow simultaneous recording of brachial and radial pulse waveforms. The time of the pulse wave upstroke was identified in real time at both recording sites, yielding brachial (t_brachial) and radial (t_radial) arrival times. Brachial–radial PWV was calculated every 3–4 s during the baseline and post-occlusion periods. Mean baseline PWV (PWVbaseline) was calculated as the average PWV during the baseline period, while mean PWV values (PWVmin_post_)were computed for each 30-second interval during the post-occlusion phase. The FMS response for each 30-second interval during the post-occlusion period was calculated as the relative change in PWV compared with baseline values (Eq. 1).

1 $$\:FMS\_min\:=\frac{(PWVmi{n}_{post}-PWVbaseline)}{PWVbaseline}\times\:100$$

### Microvascular reactivity

Microvascular reactivity was assessed using NIRS during the FMS protocol. A continuous-wave NIRS system (PortaMon, Artinis Medical Systems, The Netherlands) was used to quantify relative changes in oxygenated haemoglobin (O₂Hb) and deoxygenated haemoglobin (HHb). The NIRS sensor was positioned on the anteromedial aspect of the forearm over the flexor carpi radialis muscle, approximately 2 cm distal to the medial epicondyle of the humerus (Barbero et al. 2012). The area was marked and care was taken to ensure consistent sensor placement between measurements. The device was equipped with six light sources operating at wavelengths of 759, 760, 763, 844, and 848 nm, with an inter-optode distance of 35 mm.

Tissue saturation index (TSI) was calculated based on the modified Beer–Lambert law (Eq. 2). NIRS signals were recorded continuously throughout the protocol at a sampling frequency of 10 Hz.

2 $$\:TSI=\:\frac{{O}_{2}Hb}{({O}_{2}Hb+HHb)}\:\times\:100$$

Microvascular reactivity was assessed concurrently during the FMS protocol. Briefly, a pneumatic cuff was automatically inflated to 50 mmHg above the systolic blood pressure to induce arterial occlusion for 5 min. We used the average linear slope of TSI increase (*reoxyslope*) in the first 10 s following cuff release (during reactive hyperaemia) as a measure of microvascular reactivity, similar to established literature (McLay et al. 2016a).

### Cardiopulmonary exercise test

Following a 5-minute standardized warm-up on a cycle ergometer (Excalibur Sport, Lode, The Netherlands) at an intensity of 1 W/kg, participants performed a graded exercise test using a step-incremental protocol. The test commenced at an initial workload of 60 W, with the workload increasing by 15 W/min. Participants were instructed to maintain a cadence between 60 and 80 revolutions per minute throughout the test. Respiratory gas exchange was measured breath-by-breath using a metabolic cart (CPET, Cosmed, Italy), providing continuous measurements of oxygen uptake (V̇O₂), carbon dioxide production (V̇CO₂), and minute ventilation (V̇_E_). Heart rate was simultaneously monitored using a chest-strap heart rate monitor (H10, Polar, Finland). Participants received standardized verbal encouragement to achieve maximal effort. Attainment of maximal oxygen uptake (V̇O₂_max_) was confirmed based on established criteria, using a respiratory exchange ratio (RER) ≥ 1.10–1.15 and a ventilatory equivalent for oxygen (V̇_E_/V̇O₂) > 30–35 (Midgley et al. 2007).

Post-test data processing involved removal of aberrant breaths using the manufacturers software and export of breath-by-breath data into 5-second bins. The respiratory compensation point (RCP) along with the corresponding power output and heart rate values, were determined by an experienced evaluator (> 4 years of experience) in accordance with contemporary methodological recommendations (Keir et al. 2022).

### Exercise protocols

All exercise sessions were performed under controlled laboratory conditions (22–24 °C) on a cycle ergometer (Excalibur Sport, Lode, The Netherlands). Participants were instructed to maintain a pedalling cadence between 60 and 80 revolutions per minute during both exercise modalities. Power output at a fixed percentage of individually determined power output at RCP was used to ensure adherence to prescribed exercise domains. Additionally, heart rate was continuously monitored using a chest-strap heart rate monitor to ensure that participants exercised within the prescribed intensity domains (Coates et al. 2023).

Both protocols commenced with a 5-minute low-intensity warm-up at 1 W/kg. The HIIT session consisted of four 4-minute high-intensity intervals performed at an intensity corresponding to 100–105% of the RCP power output, interspersed with 3-minute passive recovery periods. The MICT session consisted of 30 min of continuous cycling performed at an intensity corresponding to 65% of the power output at the RCP.

### Statistical analysis

All values are presented as means ± standard deviation. Student’s t-tests were performed to check differences between sexes in baseline characteristics and primary outcomes. In case of assumption violations, appropriate non-parametric tests were performed. A within-subjects 2 × 2 repeated measures analysis of variance was conducted to test for interactions, after checking for assumption violations. Post-hoc tests with Bonferroni correction were used to assess differences between conditions. Pearson product moment correlations were computed to determine the association between the magnitude of change of different primary and secondary outcomes. Potential sex differences in response to exercise were checked using a within-subjects 2 × 2 repeated measures analysis of variance using sex as a between-subjects factor and time, as well as time x condition interactions. Statistical analysis was performed in SPSS software (IBM Statistics, IBM, USA, version: 29), as well as custom R Studio (version: 4.4.2) scripts. Statistical significance was set a priori at *p* < 0.05. A post-hoc power analysis was conducted in order to discern the observed power of the study, using GPower (3.1.9.7). We used a post-hoc achieved power calculation, using repeated-measures analysis of variance. We used our sample size, partial η^2^ of 0.196–0.336 for cfPWV and brPWV *time x condition* interaction respectively, along with an expected correlation between measurements of 0.5 and a nonsphericity correction of 1. The results indicated an observed power of 0.997–0.999, indicating the study was appropriately powered for the observed results.

## Results

Participant characteristics are presented in Table 1. We found statistically significant differences in baseline values, obtained during visit 1 between sex for weight, V̇O_2max_, and diastolic blood pressure. The difference between sexes in cfPWV was borderline significant (Table 1).

**Table 1:** Participant baseline characteristics

	Group			Male		Female			Sig.	
	*N*	M	SD	M	SD	M		SD	*p*	
Sex (M/F)	16	8/8								
Age (years)	16	24.2	3.1	25.5	3.3	22.9		2.3	0.09	
Weight (kg)	16	68	10.6	75	10	61		5.2	< 0.01*	
Body mass index (kg·m^− 2^)	16	22.5	2.1	22.9	2.1	21.9		2	0.35	
Maximal oxygen uptake (mlO_2_∙kg^− 1^·min^− 1^)	16	51.5	8.6	57.4	0.5	45.6		0.7	< 0.01*	
Diastolic blood pressure (mmHg)	16	70.4	6.0	73.4	5.5	67.2		4.2	0.025*	
Systolic blood pressure (mmHg)	16	112.4	6.6	114	6.8	110		4.7	0.26	
cfPWV (m/s)	16	6.2	0.7	6.5	0.5	5.8		0.7	0.05	
brPWV (m/s)	16	8.3	0.9	8.1	0.8	8.5		1	0.47	
FMS (%)	16	12.1	6	10	3.5	14.3		7.4	0.17^†^	
*reoxyslope* (TSI/s)	15	2.0	0.9	1.62	0.94	2.33		0.66	0.12	

* indicates statistically significant difference (*p* < 0.05) using Student’s t-test, † indicates Welch’s test for unequal variances, cfPWV – carotid-femoral pulse wave velocity, brPWV – brachial-radial pulse wave velocity, FMS – flow mediated slowing, *reoxyslope* – reactive hyperaemia reoxygenation slope

### Adherence to intervention

All included participants completed all the required testing sessions.

### Acute exercise intervention

The average heart rate between the 5th and 30th minute of MICT was 77.6 ± 5.1% HR_max_. The average heart rate in the last minute of the HIIT intervals was 83.8 ± 4.3% HR_max_, while the average maximal heart rate during the intervals was 90.8 ± 4.5% HR_max_. The maximal heart rate achieved during the last interval was 91.5 ± 4.5% HR_max_.

### Primary outcomes

Repeated measures analysis of variance revealed no significant time interactions for any measure, revealing no statistically significant changes in vascular function measures following both exercise conditions. While no interactions were statistically significant, cfPWV exhibited a slight increase following exercise (*p* = 0.086). (Table 2)

**Fig. 2:**
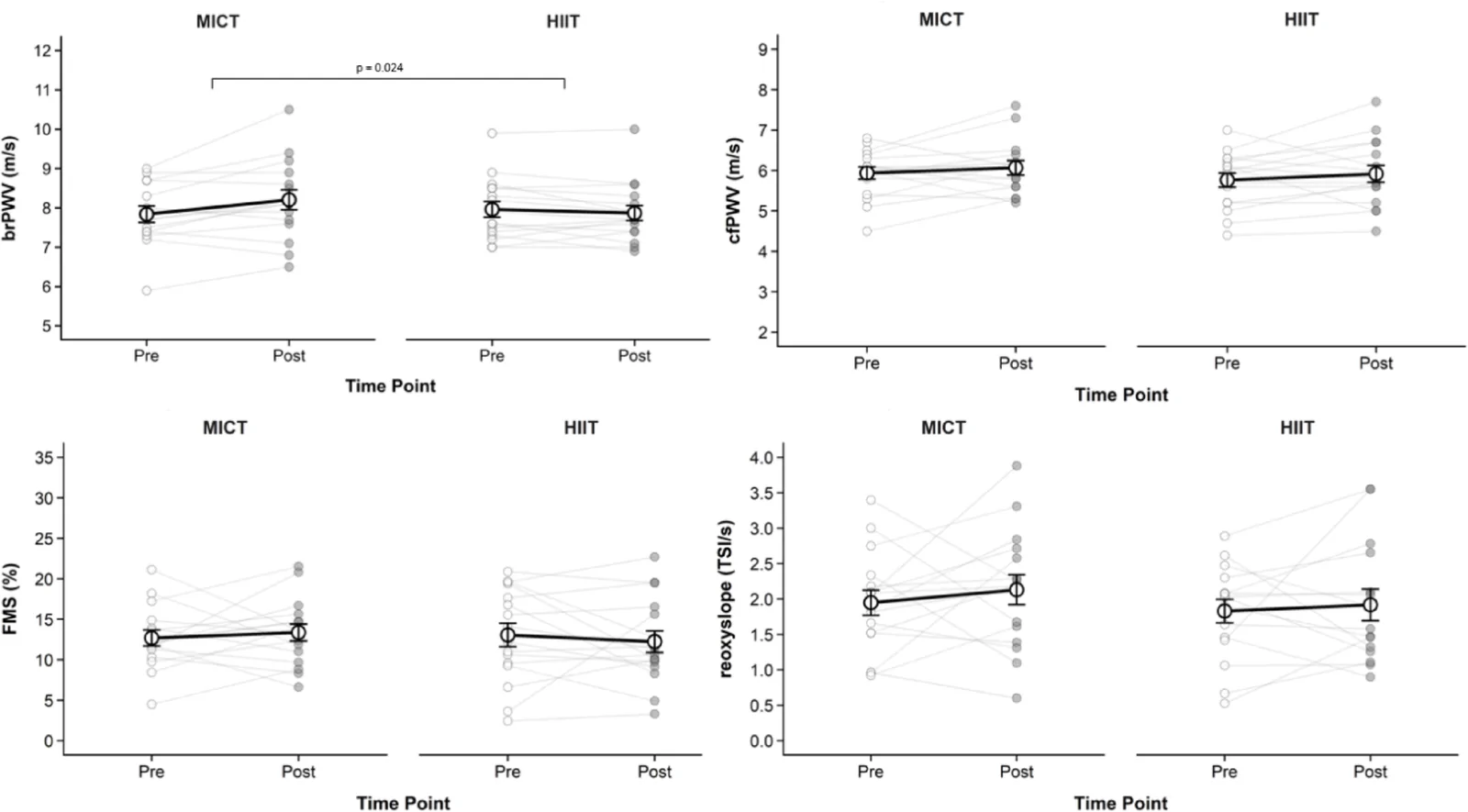
Pre to post changes in primary outcomes for both conditions. The data are presented as M ± SE and individual values. MICT – moderate intensity continuous training, HIIT – high intensity interval training, brPWV – brachial-radial pulse wave velocity, cfPWV – carotid-femoral pulse wave velocity, FMS – flow mediated slowing, reoxyslope – reactive hyperaemia reoxygenation slope

Repeated measures analysis of variance revealed a significant time-by-condition interaction for brPWV (F = 7.08, *p* = 0.024), post-hoc tests revealed a significant increase in brPWV post MICT condition (MD = 0.35 m/s) (Fig. 2). No significant time*condition interactions from pre-intervention levels were observed for cfPWV, *reoxyslope*, and FMS, indicating a differential response based on exercise intensity and modality for brPWV only, while other measures of vascular function responses remained consistent.

**Table 2:** Repeated measures analysis of variance for primary and secondary outcomes

Outcomes	Pre		Post		Mean difference	Time		Time × condition	
	M	SD	M	SD		F	*p*_adj_	F	*p*_adj_
Primary outcomes									
cfPWV (m/s)									
MICT	5.89	0.59	6.07	0.69	0.18	3.42	0.086	0.05	0.82
HIIT	5.76	0.7	5.92	0.83	0.16				
brPWV (m/s)									
MICT	7.84	0.81	8.19	1.04	0.35	2.6	0.132	7.08	0.024*
HIIIT	7.96	0.79	7.87	0.76	-0.09				
FMS									
MICT	12.69	3.95	13.37	4.14	0.68	0.005	0.94	0.92	0.35
HIIT	13.06	5.83	12.24	5.31	-0.82				
*reoxyslope* (TSI/s)									
MICT	1.95	0.71	2.13	0.84	0.18	0.46	0.51	0.05	0.83
HIIT	1.81	0.68	1.92	0.86	0.11				
Secondary outcomes									
Aix75 (%)									
MICT	7.32	6.89	17	8.08	9.72	49.3	< 0.001*	0.66	0.87
HIIT	8.02	6.57	18.1	7.96	10.12				
MAP (mm Hg)									
MICT	80.44	7.08	82	8	1.56	4.03	0.063	0.47	0.5
HIIT	79.31	7.06	82.25	4.74	2.94				
AoPP (mm Hg)									
MICT	57.31	10.32	58.88	8.39	1.56	0.18	0.68	1.04	0.32
HIIT	58.75	7.79	57.94	8.05	-0.81				

* denotes statistical significance. HIIT – high-intensity interval training, MICT – moderate-intensity continuous training, cfPWV – carotid-femoral pulse wave velocity, brPWV – brachial-radial pulse wave velocity, FMS – flow mediated slowing, *reoxyslope* – reactive hyperaemia reoxygenation slope, Aix75 – augmentation index, MAP – mean arterial pressure, AoPP – aortic pulse pressure

### Secondary outcomes

There were no significant time or time x condition interactions for pre to post both conditions for AoPP, and MAP. There was a significant time interaction for Aix75 (F = 49.3, *p* < 0.001), indicating an increase following both conditions (F = 49.3, *p* < 0.001). While not significant (*p* = 0.063), MAP indicated a slight increase following both conditions (Table 2).

We ran a correlation analysis for the magnitude of response of each selected primary outcome for pre- to post each condition (∆cfPWV_MICT vs. ∆cfPWV_HIIT; (∆brPWV_MICT vs. ∆brPWV_HIIT, etc.). No significant interactions were observed indicating that the magnitude of response to MICT did not correlate with the magnitude of response to HIIT. To address the potential sex-specific vascular responses, a repeated measures of variance analysis using sex as between-subjects factor was employed. The results show no statistically significant differences in time x sex or time x condition x sex interaction for any of the outcomes (Table 3), indicating a uniform vascular function response regardless of sex.

**Table 3:** Repeated measures analysis of variance by sex for vascular function response to both conditions

Outcome	Time x sex			Time × condition × sex		
	F (1,13)	*p*	η_*p*_^2^	F (1,13)	*p*	η_*p*_^2^
cfPWV	1.3	0.276	0.091	0.64	0.44	0.047
brPWV	1.41	0.257	0.098	0.256	0.622	0.019
FMS	0.03	0.86	0.002	1.96	0.18	0.123
reoxyslope	0.064	0.804	0.005	0.82	0.38	0.06

cfPWV – carotid-femoral pulse wave velocity, brPWV – brachial-radial pulse wave velocity, FMS – flow mediated slowing, reoxyslope – reactive hyperaemia reoxygenation slope

## Discussion

The primary aim of this study was to evaluate the acute responses of multiple vascular function measures to MICT and HIIT in healthy, physically active young adults with no known CVD risk factors. We used a multi-segmental approach, assessing central and peripheral arterial stiffness, as well as FMS and microvascular reactivity following a vascular occlusion test.

Our main results highlight a differential response of central and peripheral arterial stiffness, identifying exercise intensity and modality as key modifiable factors influencing peripheral arterial stiffness following acute exercise. Central arterial stiffness did not exhibit a significant change following exercise, regardless of condition, whereas peripheral arterial stiffness exhibited a differential response. Peripheral arterial stiffness increased following MICT and exhibited no change following HIIT. There were no significant changes in the microvascular reactivity and FMS following both conditions, indicating a stability in response regardless of exercise intensity.

### Central arterial stiffness

Central arterial stiffness exhibited no significant differences between the two conditions, suggesting a uniform response. A review by Mutter et al. (2017) suggested a tendency towards increased cfPWV immediately following exercise with a gradual decrease below pre-intervention levels in the period 5–30 min post-intervention, even though the responses in the included studies were not uniform. Exercise mode seems to influence the acute response to exercise, with differential response to aerobic, compared to resistance exercise (Heffernan et al. 2007). This response is akin to numerous responses observed in human physiology and represents acute deterioration in the function in response to the stressor (exercise), followed by a return to baseline values and a potential supercompensation, similar to responses observed for FMD (Dawson et al. 2013, 2018). Interestingly, another meta-analysis by Pierce et al. (2018) did not reveal significant changes in cfPWV following acute AE. Part of the reason for their lack of observed change might be explained precisely by the timing of the measurements, as well as possible differences in response based on exercise intensity and modality. In light of the limited research on the effects of exercise intensity on these measures, our results show no change in cfPWV following both exercise conditions, suggesting that the response in central arteries is not dependent on the specific exercise intensity. While systematic reviews of the literature generally don’t report significant effects of exercise on central arterial stiffness, a significant heterogeneity in responses is present. Lefferts et al. (2022) found increased cfPWV following exercise in hypertensive and normotensive individuals. Similarly, Rakobowchuk et al. (2009), reported an increase in central arterial stiffness, albeit following sprint exercise. Working upon this framework, our results show a uniform response of cfPWV, suggesting that this response is independent of exercise intensity and is the same in young, healthy, individuals with optimal blood pressure as in the hypertensive patients presented in the aforementioned studies.

The physiological mechanisms for this response may lie in the fact that arterial stiffness is a measure of both the mechanical properties of the arteries, as well as their vascular tone. Numerous stressors may exhibit an influence on arterial stiffness, including heart rate, sympathetic activity, viscosity, as well as blood pressure, and vasomotor tone. As evidenced by Naka et al. (2003) post-exercise changes in arterial stiffness may not be attributed to alterations in heart rate or blood viscosity. Another study demonstrated a direct association between sympathetic activity and aortic stiffness (Nardone et al. 2018), supporting the notion that acute changes in arterial stiffness may result from heightened sympathetic activation and elevated blood pressure during exercise, which together influence arterial stiffness and vasomotor tone, leading to increases or decreases depending on the vascular segment assessed. In these terms, cfPWV reflects the systemic response of aortic stiffness. The aorta exhibits limited vasomotor capacity due to the predominance of elastic lamellae over smooth muscle cells in the tunica media, contrary to peripheral arteries (Hosoda et al. 1984; Silver et al. 2003). Therefore, a slight increase in cfPWV immediately following exercise is likely modulated by sympathetic activity and load-dependent mechanical stiffening of the arterial wall. Different exercise modalities present markedly different types of stress on the vessel wall. In terms of our study, MICT elicits a sustained increase in laminar flow, whereas HIIT generates transient turbulent shear patterns and higher peak shear stress during intervals, interspersed with lower values during recovery (Tinken et al. 2009; Shi et al. 2022). Even though these exercise modalities elicit distinct shear stress patterns, we did not observe differential changes in cfPWV, providing further evidence, that factors other than shear stress primarily regulate the acute cfPWV response following exercise. There was a borderline significant difference in baseline cfPWV values between males and females, with females exhibiting reduced central arterial stiffness compared to males (*p* = 0.05). Regardless of baseline differences between sexes, the response to exercise was uniform for both sexes.

### Peripheral arterial stiffness

We observed a differential peripheral arterial stiffness response, with an increase following MICT and no change following HIIT. It is difficult to compare this result to existing literature, as most used brachial-ankle PWV, as opposed to brPWV used in our study. We chose brPWV specifically, to observe systemic changes in the peripheral part of the vascular system, excluding measures influenced by actively perfused lower-limb arterial segments. Nevertheless, numerous studies observed decreases in brachial-ankle PWV following exercise (Zang et al. 2022; Huang et al. 2023; Feng et al. 2025). However, these discrepancies might be attributed to the differential effect of acute arterial stiffness changes in upper versus lower arterial segments. Mutter et al. (2017) have shown that central and upper-segment arterial stiffness increases immediately following exercise, while lower-segment arterial stiffness decreases immediately following exercise. Combined with the different timing of these measurements, it is difficult to contextualize our results within the broader scope of the literature. Mechanistically, this response might be interpreted as a difference in systemic vasomotor response following MICT compared to HIIT. Peripheral arteries are inherently more responsive to vasodilatory substances and exhibit a greater capacity for vasomotion (Thijssen et al. 2012). The increased brPWV following MICT might reflect systemic vasoconstriction resulting from blood flow redirection toward active muscle beds, coupled with increased sympathetic activity and elevated blood pressure during exercise. Conversely, the attenuation of brPWV following HIIT might reflect a pronounced systemic vasodilatory response, driven by increased shear stress and the subsequent upregulation of endothelial nitric oxide synthase and nitric oxide immediately following exercise. This may nullify the increases associated with heightened sympathetic activity and blood pressure resulting from elevated shear stress and sympathetic activity. Nevertheless, the observed effects were not statisitcally significant.

### Flow mediated slowing

No changes were observed for FMS when looking at the whole sample, indicating no significant alteration following either condition. In the present study, FMS was quantified using both the maximal change in PWV following occlusion (FMS_min) and the greatest mean change in PWV within a 30-second post-occlusion time window, as proposed by Van Criekinge et al. (2025). However, neither methodological approach revealed significant changes and differences between conditions. We were not able to adequately quantify changes in FMS, and a critical evaluation of the reliability of this measure is warranted before drawing definitive conclusions. To the best of our knowledge, no studies to date have investigated the acute FMS response following a bout of physical exercise. Several studies have reported correlation between FMS and FMD (Ellins et al. 2016; Pereira et al. 2018; Lößner et al. 2023). However, considerable caution is warranted when using FMS as a proxy of FMD, especially following acute exercise. Generally, the results on FMS reliability are heterogeneous, with some studies reporting poor repeatability of FMS compared to FMD (Marôco et al. 2022), whereas others have demonstrated good repeatability and reproducibility (Ellins et al. 2016; Marôco et al. 2023). Based on the magnitude and heterogeneity of responses observed in our study, we cannot confidently propose FMS as a reliable measure for approximating vasodilatory response to reactive hyperaemia following acute exercise in this cohort. Additional methodological studies are therefore required to establish the sensitivity, reliability, and physiological validity of oscillometric FMS for the assessment of acute exercise-induced vascular responses.

### Microvascular reactivity

Microvascular reactivity exhibited heterogeneous responses, similar to those observed for FMS. Based on our findings, microvascular reactivity remained relatively stable following different intensities of exercise. Current literature has mainly used microvascular reactivity in a cross-sectional design to differentiate between healthy and clinical populations (Dipla et al. 2017) as well as individuals with different training statuses (McLay et al. 2016b). Interestingly, a recent study by Landers-Ramos et al. (2026) has found increased microvascular reactivity following a submaximal cycling exercise bout, while the values remained stable following a maximal cycling test. In terms of baseline reoxyslope values, our results mirror those of Landers-Ramos et al. (2026), indicating that the reoxyslope values of about 2% TSI/s are to be expected when examining healthy, physically active young adults. We have no been able to show a consistent response of microvascular reactivity following MICT or HIIT. Thus, our results suggest that microvascular reactivity remains constant irrespective of mode and intensity of AE within the range of intensities observed in this study. Our results in conjunction with the current available data show that reoxyslope is influenced by participants’ health status, as well as training status. While the baseline values are influenced by these factors, the response to exercise was stable following exercise in moderate as well as high-intensity exercise domain. The heterogeneity in response leads us to believe that microvascular reactivity immediately post-exercise might also be modulated by other factors, in addition to exercise.

### Limitations

Due to the methodological approach, several limitations have to be acknowledged. We were unable to comprehensively control for the activity of the participants between visits, nor the nutritional assessment and its timing before the measurement, beyond standardized verbal recommendations. Additionally, it was not possible to assess all the vascular function measures simultaneously, limiting our ability to evaluate the time dependency of these measures. Nevertheless, all measurements were conducted within 5–15 min period post exercise cessation, which falls within the range where these measures should remain stable according to Mutter et al. (2017). Even though this methodological approach precluded us from measuring at exact time points, it enabled us to evaluate different parts of the vascular system within one testing session, therefore this limitation can also be considered a strength of the study. Finally, we did not quantify shear stress during exercise, therefore any implications on the difference in response due to shear stress pattern are inferred from the existing literature.

## Conclusions

In conclusion, both MICT and HIIT appear to elicit homogenous responses pertaining to measures of central arterial stiffness and haemodynamics. However, peripheral arterial stiffness exhibited a differential response based on exercise intensity and modality. Peripheral arterial stiffness increased following MICT, while no change was observed following HIIT. Microvascular reactivity and FMS did not result in meaningful changes following each condition. Future studies should further examine the type and time-dependency of the acute effect of MICT and HIIT on both active and inactive segments of the vascular system, as well as their potential for long-term vascular adaptations.

## Data Availability

All data acquired for this study are available from the corresponding author upon reasonable request. The data can be accessed at: https://repozitorij.uni-lj.si/IzpisGradiva.php?id=186603 with the permanent ID: http://hdl.handle.net/20.500.12556/RUL-186603.

## Abbreviations

AE: Aerobic exercise
Aix: Augmentation index
AoPP: Aortic pulse pressure
brPWV: Brachial-radial pulse wave velocity
cfPWV: Carotid-femoral pulse wave velocity
CVD: Cardiovascular disease
FMD: Flow mediated dilation
FMS: Flow mediated slowing
HHb: Deoxygenated haemoglobin
HIIT: High-intensity interval training
HR: Heart rate
MAP: Mean arterial pressure
MICT: Moderate-intensity continuous training
NIRS: Near-infrared spectroscopy
O_2_Hb: Oxygenated haemoglobin
PWV: Pulse wave velocity
PWVbaseline: Baseline brachial-radial pulse wave velocity
PWVmin_post_: Minimally attained brachial-radial pulse wave velocity
RCP: Respiratory compensation point
reoxyslope: Reoxygenation slope during reactive hyperaemia
RER: Respiratory exchange ratio
TSI: Tissue saturation index
V̇CO_2_: Carbon dioxide production
V̇O_2_: Oxygen uptake
V̇O_2max_: Maximal oxygen uptake

## References

[CR1] Armstrong T, Bull F (2006) Development of the World Health Organization Global Physical Activity Questionnaire (GPAQ). J Public Health (Bangkok) 14:66–70. 10.1007/S10389-006-0024-X

[CR2] Barbero M, Merletti R, Rainoldi A (2012) Atlas of muscle innervation zones. Atlas Muscle Innervation Zones. 10.1007/978-88-470-2463-2

[CR3] Coates AM, Joyner MJ, Little JP et al (2023) A perspective on high-intensity interval training for performance and health. Sports Med 53:85–96. 10.1007/s40279-023-01938-6 PMC10721680 37804419

[CR5] Dawson EA, Green DJ, Timothy Cable N, Thijssen DHJ (2013) Effects of acute exercise on flow-mediated dilatation in healthy humans. J Appl Physiol 115:1589–1598. 10.1152/japplphysiol.00450.2013 24030665

[CR4] Dawson EA, Cable NT, Green DJ, Thijssen DHJ (2018) Do acute effects of exercise on vascular function predict adaptation to training? Eur J Appl Physiol 118:523–530. 10.1007/s00421-017-3724-8 PMC5805792 29234916

[CR6] Dipla K, Triantafyllou A, Koletsos N et al (2017) Impaired muscle oxygenation and elevated exercise blood pressure in hypertensive patients. Hypertension 70:444–451. 10.1161/HYPERTENSIONAHA.117.09558 28607132

[CR7] Ellins EA, New KJ, Datta DBN et al (2016) Validation of a new method for non-invasive assessment of vasomotor function. Eur J Prev Cardiol 23:577–583. 10.1177/2047487315597210 26209709

[CR8] Feng W, Yu W, Zhao Z et al (2025) Acute effects of high-intensity interval exercise and moderate-intensity continuous training on arterial stiffness and endothelial function in hypertension: A crossover trial. Sci Rep 15:37086. 10.1038/s41598-025-21045-y PMC12550041 41131037

[CR9] Heffernan K, Collier S, Kelly E et al (2007) Arterial stiffness and baroreflex sensitivity following bouts of aerobic and resistance exercise. Int J Sports Med 28:197–203. 10.1055/s-2006-924290 17024636

[CR10] Hosoda Y, Kawano K, Yamasawa F et al (1984) Age-dependent changes of collagen and elastin content in human aorta and pulmonary artery. Angiology 35:615–621. 10.1177/000331978403501001 6497045

[CR11] Huang Z, Chen G, Wang X et al (2023) The effect of acute aerobic exercise on arterial stiffness in individuals with different body fat percentages: a cross-sectional study. Front Cardiovasc Med 9:1072191 10.3389/fcvm.2022.1072191 PMC9874153 36712258

[CR12] Keir DA, Iannetta D, Mattioni Maturana F et al (2022) Identification of non-invasive exercise thresholds: methods, strategies, and an online app. Sports Med 52:237–255. 10.1007/S40279-021-01581-Z 34694596

[CR13] Landers-Ramos RQ, Silva T, Dobrosielski DA, Knuth ND (2026) Examination of post-exercise microvascular reactivity in healthy adults. Eur J Appl Physiol 126:375–386. 10.1007/s00421-025-05906-y PMC12881111 40694131

[CR14] Lefferts WK, Weiner CM, Mascone SE et al (2022) Influence of sex and presence of cardiovascular risk factors on relations between cardiorespiratory fitness and cerebrovascular hemodynamics. J Appl Physiol 133:1019–1030 10.1152/japplphysiol.00371.2022 PMC9576163 36074924

[CR15] Lößner C, Multhaup A, Lehmann T et al (2023) Sonographic flow-mediated dilation imaging versus electronic endocheck flow-mediated slowing by VICORDER in pregnant women—a comparison of two methods to evaluate vascular function in pregnancy. J Clin Med 2023 12:121719. 10.3390/JCM12051719 PMC10003587 36902503

[CR16] Mancia G, Kreutz R, Brunström M et al (2023) 2023 ESH Guidelines for the management of arterial hypertension The Task Force for the management of arterial hypertension of the European Society of Hypertension. J Hypertens 41:1874–2071. 10.1097/HJH.0000000000003480 37345492

[CR17] Marôco JL, Pinto M, Santa-Clara H et al (2022) Flow-mediated slowing shows poor repeatability compared with flow-mediated dilation in non-invasive assessment of brachial artery endothelial function. PLoS ONE 17:e0267287. 10.1371/JOURNAL.PONE.0267287 PMC9129018 35609038

[CR18] Marôco JL, Silvestre T, Arrais I et al (2023) Intra and inter-rater repeatability of brachial artery ultrasound estimates of flow-mediated slowing and flow-mediated dilation. PLoS ONE 18. 10.1371/JOURNAL.PONE.0287759 PMC10306196 37379344

[CR19] Mattioni Maturana F, Martus P, Zipfel S, Nieß AM (2021) Effectiveness of HIIE versus MICT in improving cardiometabolic risk factors in health and disease: a meta-analysis. Med Sci Sports Exerc 53:559–573. 10.1249/MSS.0000000000002506 32890201

[CR20] McKay AKA, Stellingwerff T, Smith ES et al (2022) Defining training and performance caliber: a participant classification framework. Int J Sports Physiol Perform 17:317–331. 10.1123/IJSPP.2021-0451 34965513

[CR21] McLay KM, Fontana FY, Nederveen JP et al (2016a) Vascular responsiveness determined by near-infrared spectroscopy measures of oxygen saturation. Exp Physiol 101:34–40. 10.1113/EP085406 26498127

[CR22] McLay KM, Gilbertson JE, Pogliaghi S et al (2016b) Vascular responsiveness measured by tissue oxygen saturation reperfusion slope is sensitive to different occlusion durations and training status. Exp Physiol 101:1309–1318. 10.1113/EP085843 27430198

[CR23] Midgley AW, McNaughton LR, Polman R, Marchant D (2007) Criteria for determination of maximal oxygen uptake: a brief critique and recommendations for future research. Sports Med 37:1019–1028. 10.2165/00007256-200737120-00002/FIGURES/1 18027991

[CR24] Mutter AF, Cooke AB, Saleh O et al (2017) A systematic review on the effect of acute aerobic exercise on arterial stiffness reveals a differential response in the upper and lower arterial segments. Hypertens Res 40:146–172 10.1038/hr.2016.111 27733765

[CR25] Naka KK, Tweddel AC, Parthimos D et al (2003) Arterial distensibility: acute changes following dynamic exercise in normal subjects. Am J Physiol Heart Circ Physiol 284:H970–H978. 10.1152/ajpheart.00529.2002 12433655

[CR26] Nardone M, Incognito AV, Millar PJ (2018) Evidence for pressure-independent sympathetic modulation of central pulse wave velocity. J Am Heart Assoc 7. 10.1161/JAHA.117.007971 PMC5850264 29378730

[CR27] Paravlic AH, Iskra S, Abazovic E et al (2025) Comparing aerobic interval training with other forms of physical exercise for brachial artery endothelial function improvement: a systematic review and network meta-analysis of randomized controlled trials. Sports Med Open 11:133. 10.1186/s40798-025-00929-3 PMC12638609 41269584

[CR28] Pereira T, Almeida A, Conde J (2018) Flow-mediated slowing as a methodological alternative to the conventional echo-tracking flow-mediated dilation technique for the evaluation of endothelial function: a preliminary report. Mayo Clin Proc Innov Qual Outcomes 2:199–203. 10.1016/j.mayocpiqo.2018.02.002 PMC6124332 30225450

[CR29] Pierce DR, Doma K, Leicht AS (2018) Acute effects of exercise mode on arterial stiffness and wave reflection in healthy young adults: a systematic review and meta-analysis. Front Physiol 9:73 10.3389/fphys.2018.00073 PMC5816907 29487535

[CR30] Rakobowchuk M, Stuckey MI, Millar PJ et al (2009) Effect of acute sprint interval exercise on central and peripheral artery distensibility in young healthy males. Eur J Appl Physiol 105:787–795. 10.1007/s00421-008-0964-7 19125283

[CR31] Saz-Lara A, Cavero-Redondo I, Álvarez-Bueno C et al (2021) The acute effect of exercise on arterial stiffness in healthy subjects: a meta-analysis. J Clin Med 10:291. 10.3390/jcm10020291 PMC7831005 33466830

[CR32] Schmalenberger KM, Tauseef HA, Barone JC et al (2021) How to study the menstrual cycle: Practical tools and recommendations. Psychoneuroendocrinology 123:104895. 10.1016/j.psyneuen.2020.104895 PMC8363181 33113391

[CR33] Shenouda N, Priest SE, Rizzuto VI, MacDonald MJ (2018) Brachial artery endothelial function is stable across a menstrual and oral contraceptive pill cycle but lower in premenopausal women than in age-matched men. Am J Physiol Heart Circ Physiol 315:H366–H374. 10.1152/ajpheart.00102.2018 29727219

[CR34] Shi W, Liu H, Cao L et al (2022) Acute effect of high-intensity interval exercise on vascular endothelial function and possible mechanisms of wall shear stress in young obese males. Front Physiol 13. 10.3389/fphys.2022.966561 PMC9523906 36187796

[CR35] Silver FH, Snowhill PB, Foran DJ (2003) Mechanical behavior of vessel wall: a comparative study of aorta, vena cava, and carotid artery. Ann Biomed Eng 31:793–803. 10.1114/1.1581287 12971612

[CR36] Thijssen DHJ, Cable NT, Green DJ (2012) Impact of exercise training on arterial wall thickness in humans. Clin Sci 122:311–322. 10.1042/CS20110469 PMC3233305 22150253

[CR37] Tinken TM, Thijssen DHJ, Hopkins N et al (2009) Impact of shear rate modulation on vascular function in humans. Hypertension 54:278–285. 10.1161/HYPERTENSIONAHA.109.134361 PMC3012006 19546374

[CR38] Tordi N, Mourot L, Colin E, Regnard J (2010) Intermittent versus constant aerobic exercise: effects on arterial stiffness. Eur J Appl Physiol 108:801–809. 10.1007/s00421-009-1285-1 20187285

[CR39] Van Criekinge H, Renier M, Decorte E et al (2025) Defining abnormal flow-mediated slowing of brachial-radial pulse wave velocity, a noninvasive vasoreactivity test. J Hypertens 43. 10.1097/HJH.0000000000004146 41031703

[CR40] Williams JS, Dunford EC, Macdonald MJ (2020) Impact of the menstrual cycle on peripheral vascular function in premenopausal women: systematic review and meta-analysis. Am J Physiol Heart Circ Physiol 319. 10.1152/ajpheart.00341.2020.-Fluctuations 33064553

[CR41] Zang Y, Ding X, Zhao MX et al (2022) Arterial stiffness acute changes following aerobic exercise in males with and without hypertension. J Clin Hypertens (Greenwich) 24:430–437 10.1111/jch.14461 PMC8989744 35285576

